# Draft Genome Sequence of Salmonella enterica Serovar Enteritidis from Jos, Plateau State, Nigeria

**DOI:** 10.1128/MRA.00576-21

**Published:** 2021-12-02

**Authors:** Oladele Oludotun Olubusola, Ameji Negedu Onogu, Jambalang Alexander Ray, Olufunmilola Olorundare, Grace Chiemeka Ezemokwe, Uchenna Precious Nnadi, Francis Agwom, Oluwatoyin Ruth Morenikeji, Gimba Nanko, Anayochukwu Chibuike Ngene, John Chineye Aguiyi, Sebastian Leptihn, Nnaemeka Emmanuel Nnadi

**Affiliations:** a Department of Veterinary Medicine, Surgery and Radiology, University of Jos, Jos, Nigeria; b Bacteria Research Department, National Veterinary Research Institute, Vom, Nigeria; c Department of Pest Management Technology, Federal College of Forestry, Jos, Nigeria; d Department of Microbiology, Africa Centre of Excellence in Phytomedicine Research and Development (ACEPRD), University of Jos, Jos, Nigeria; e Department of Small Animal Medicine, Ahmadu Bello University, Zaria, Nigeria; f Department of Pharmaceutical & Medicinal Chemistry, Faculty of Pharmaceutical Sciences, University of Jos, Jos, Nigeria; g Department of Microbiology, College of Natural Sciences, Michael Okpara University of Agriculture, Umudike, Abia State, Nigeria; h Zhejiang University-University of Edinburgh Institute, Zhejiang University, Haining, China; i Department of Infectious Diseases, Sir Run Run Shaw Hospital, School of Medicine, Zhejiang University, Hangzhou, China; j Infection Medicine, Biomedical Sciences, Edinburgh Medical School, College of Medicine and Veterinary Medicine, The University of Edinburgh, Edinburgh, United Kingdom; k Department of Microbiology, Faculty of Natural and Applied Sciences, Plateau State University, Bokkos, Nigeria; University of Maryland School of Medicine

## Abstract

Salmonella enterica serovar Enteritidis causes the highest incidence of human salmonellosis infections. Here, we describe the whole-genome sequence and annotation of Salmonella enterica serovar Enteritidis strain 1145s, isolated in Nigeria. The strain has a genome of 4.57 Mb with a GC content of 52% and contains one plasmid.

## ANNOUNCEMENT

Salmonella enterica serovar Enteritidis causes the highest incidence of human salmonellosis infections (1). It is considered an emerging pathogen, with changing epidemiology over the last 20 years (2). Salmonella Enteritidis is a zoonotic disease associated with poultry (3). In this study, the genome of Salmonella enterica serovar Enteritidis strain 1145s, recovered from a poultry farm in Jos (Plateau State, Nigeria), was sequenced and annotated.

Strain 1145s was isolated from chicken organs (liver, heart, spleen, and kidney) from a chicken with salmonellosis. Samples of these organs were streaked onto 5% blood agar and MacConkey agar plates, which were incubated aerobically at 37°C for 24 h. Tiny colonies growing on MacConkey agar contained cells that were Gram negative when stained. Biochemical tests such as starch hydrolysis, oxidase, catalase, indole, and sugar fermentation tests (4) were performed, identifying the strain as Salmonella.

For sequencing, DNA extraction was performed using a DNeasy blood and tissue kit (Qiagen), following the manufacturer’s instructions. A sequencing library was prepared using the Nextera XT DNA sample preparation kit (Illumina), following the manufacturer’s protocol. Whole-genome sequencing was performed at the Antimicrobial Resistance Laboratory, Microbial Genomics Reference Laboratory, CIDMLS, ICPMR, Westmead Hospital (Australia), using the Illumina NextSeq 500 platform. A total of 650 Mbp of 2 × 150-bp paired-end reads were generated, with a coverage of 152× for the genome. The raw data were trimmed using fastp (5) and assembled using SPAdes v3.13.0 (6). The genome completeness was determined using QUAST v5.0.2 (7) and BUSCO (8). Genome annotation was performed using the NCBI Prokaryotic Genome Annotation Pipeline (PGAP) v5.2 (9). Salmonella serotyping was predicted using the SeqSero v2.0 software tool (6). Salmonella pathogenicity islands (SPIs) were identified using the SPIFinder v1.0 online tool (https://cge.cbs.dtu.dk/services/SPIFinder). Default parameters were used for the tools.

PlasmidFinder v2.1 (https://cge.cbs.dtu.dk/services/PlasmidFinder/) was used to determine the presence of plasmids (10). The presence of antimicrobial resistance (AMR) genes was investigated using ResFinder v3.1 with default settings (https://cge.cbs.dtu.dk/services/ResFinder/). The results were correlated with the phenotypic observations obtained using antimicrobial susceptibility testing (AST). S. Enteritidis 1145s has a total of 36 contigs in a genome of 4.57 Mb, with an average GC content of 52% and an *N*_50_ value of 304,938 bp. The strain contains one plasmid identical to pSPCV (Salmonella enterica subsp. *enterica* serovar Paratyphi C; GenBank accession number CP000858) (11). Strain 1145s is 98.8% complete based on the benchmarking universal single-copy ortholog (BUSCO) values (containing 122 of the 124 total BUSCO genes).

### Data availability.

The complete genome sequences of the strain have been deposited at the NCBI GenBank database under the accession number JAGDDT000000000, the BioProject accession number PRJNA713960, and the BioSample accession number SAMN18275554.
